# Nutrient Content, Functional Properties and Acceptability of Rock Buns Formulated From Freeze-Dried Detroit Dark Red Beetroot Pomace Flour

**DOI:** 10.1155/ijfo/9986191

**Published:** 2025-07-26

**Authors:** Isaac Amoah, Ransford Opoku Asante, Rose Attakora, Abdallah Zaidan Mohammed, Marina Aferiba Tandoh, Charles Diako

**Affiliations:** ^1^Department of Biochemistry and Biotechnology, Kwame Nkrumah University of Science and Technology, Kumasi, Ghana; ^2^School of Food Technology and Natural Sciences, Massey University, Palmerston North, New Zealand

**Keywords:** bakery product, beetroot pomace, functional properties, proximate analysis, rock buns, sensory evaluation, upcycling

## Abstract

Beetroot pomace is an underutilised food by-product obtained from the processing of beetroots. However, its rich source of nutrients makes it a potential ingredient for utilisation in rock bun development. The aim of the study was to investigate the nutrient composition and functional properties of freeze-dried beetroot pomace and wheat composite flours, as well as the sensory acceptability of rock buns formulated from these flours. Rock buns were formulated with 95:5, 90:10, 85:15 and 80:20 for wheat flour:freeze-dried beetroot pomace flour (BPF) and 100:0 for the control, respectively. Functional properties and proximate analysis of the flours were determined using standard methods. Sensory evaluation was carried out using a 100 mm visual analogue scale. One-way ANOVA was used to determine significant differences in the mean of the parameters evaluated. Principal component analysis and agglomerative hierarchical clustering exploiting Ward linkage and Euclidean distances were used to establish a visual relationship between the flour samples and some outcome variables. The nutrient composition of composite flour enriched with 20% of BPF showed high fibre, protein and ash content of 1.79%, 11.71% and 2.19%, respectively, compared to the control sample. The swelling power, oil absorption capacity and water absorption capacity increased with increased incorporation of BPF, whilst dispersibility and solubility decreased. The sensory acceptability of the rock buns enriched with 5% and 10% freeze-dried BPF was comparable to the control rock buns formulated from wheat flour only. Rock buns enriched with 10% freeze-dried BPF are nutrient-dense and can compete with control rock buns commercially.

## 1. Introduction

Rock buns are one of the most commonly consumed bakery products in Ghana. They are traditionally formulated from refined wheat flour, salt, sugar and margarine, which are inferior in nutrients such as fibre, but are energy-dense and contain rapidly digestible carbohydrates [1]. Increased and regular consumption of foods with poor nutrient quality such as those with high salt, refined sugar and depleted fibre content due to low fruit and vegetable content is an essential risk factor for Type 2 diabetes and hypertension development [2, 3]. In Ghana, hypertension, for example, is currently the leading cause of death [4] with a national prevalence of 27% [5]. Additionally, the prevalence of Type 2 diabetes is expected to increase from 2.6% in 2021 to 3.1% in 2030 and 3.3% in 2045, respectively, in adults (aged 20–79 years) [6]. A strategic and cost-effective approach that could be exploited to improve the nutrient density of commonly consumed snacks such as rock buns includes reformulation of the base refined wheat flour with other nutrient-dense ingredients of plant-based origin [7].

In the past decade, the concept of enriching commonly consumed nutrient-depleted staples with plant-based agroprocessing by-products, commonly termed upcycling, has become a common phenomenon in the food product development area [8]. This strategy has gained popularity, especially because it feeds into the United Nations Development Goals 2 and 12, which prioritise zero hunger and sustainable processing of food materials [9]. In the past, we enriched rock buns with pineapple and carrot pomaces and showed that rock buns enriched with 15% carrot and 5% pineapple pomaces recorded significantly higher fibre (2.85%) content compared to a control sample which recorded 0.42% [10]. Beetroot pomace, the by-product obtained after beetroot processing into juice, is another important food by-product that is mainly being discarded but could be exploited for rock bun development to reduce the environmental impact of its disposal.

Beetroot pomace is a prominent source of nutrients and health-promoting compounds including potassium, calcium, magnesium, flavonoid, phenolics, betacyanin and betaxanthin [11, 12]. Consequently, its application in food products such as bread, cakes, biscuits, cookies, pasta, noodles and extruded products to enhance nutritional density and health-promoting benefits of these products [13, 14] has been reported but not in rock buns. The pomace is usually processed into flour, extract and puree prior to enrichment with other food products [13]. Enrichment of cookies with beetroot pomace flour (BPF) at substitution levels of 5%, 10%, 15%, 20% and 25% resulted in increased fibre (1.20%–2.92%) composition compared to the control cookie [14].

Despite the high nutrient density of beetroot pomace, its high moisture content of 86.8% [11] renders it susceptible to deterioration. Several processing methods such as freeze-drying, spray drying and vacuum microwave drying could be exploited for beetroot pomace processing for food product enrichment [15]. Freeze-drying of plant-based food by-products remains one of the most common drying methods due to its ability to retain heat-labile nutrients and bioactive compounds compared to the conventional oven drying method [16]. Freeze-drying, however, may affect the functional properties of these plant-based food by-products such as the water absorption capacity (WAC), oil absorption capacity (OAC) and swelling power (SP) and ultimately impact its technological properties. Also, the higher fibre content of BPF may adversely impact the technological properties of a composite dough, leading to gluten network dilution in the dough as observed for blackcurrant pomace in a previous study [17]. This may subsequently affect the overall sensory properties of the rock buns when BPF is utilised in composite preparation with refined wheat flour for rock bun development [18]. Potential adverse effects following the enrichment of rock buns with BPF especially beyond the acceptable concentration may include negative physical qualities of the rock buns and sensory properties [8]. Consequently, investigating the functional properties of the BPF used in composite preparation with refined wheat flour is important. Additionally, there is a need to ensure adequate utilisation of BPF in rock bun development to deliver products with desirable sensory properties. The study is aimed at investigating the nutrient composition and functional properties of freeze-dried beetroot pomace and wheat composite flours and the sensory acceptability of its formulated rock buns.

## 2. Materials and Methods

### 2.1. Source of Materials

Fresh Detroit dark red beetroots were obtained from the University of Cape Coast agricultural farm in the Central Region of Ghana. The ingredients for the preparation of the rock buns (wheat flour, margarine, salt, sugar, baking powder, eggs, nutmeg and vanilla essence) were purchased from the Kejetia Market, a popular market in the Ashanti Region enclave of Ghana. This research was carried out at the Departments of Biochemistry and Biotechnology and Food Science and Technology Laboratories of the Kwame Nkrumah University of Science and Technology (KNUST), Kumasi, Ghana.

### 2.2. Sample Preparation

The beetroots were rinsed under running water to get rid of dirt and blanched in water at 60°C for 8 min to soften the beetroot tissues and reduce the microbial load of the raw beetroots. The beetroot was peeled using a stainless steel knife and diced into shapes of about 3 mm. A 100 g of the diced beetroot from each batch was weighed into a juice extractor, and the pomace obtained after the juice extraction process was collected. Freeze-drying was employed as the most appropriate method for the beetroot pomace processing for this study, as a preliminary trial revealed a well-dried pomace from freeze-drying than the oven drying method. The pomace was freeze-dried using a freeze-dryer (YK-118-50, True Ten Industrial Co., Ltd., China) within 48 h until constant weight was gained. The pressure during sublimation was maintained at 20 Pa and reduced to 5 Pa during desorption. The initial temperature during sublimation was set at −25°C, and this was increased gradually up to −40°C during desorption. The freeze-dried beetroot pomace was pulverised using a mixer grinder electric blender (MX-AC210, Panasonic, Japan). The flour obtained was passed through a sieve with 425 *μ*m pore size, and the BPF obtained was packaged into a double zipper high-density Ziploc bag and stored at room temperature until ready for further analysis.

### 2.3. Proximate Analysis of the Beetroot Pomace

The proximate analysis carried out on the beetroot pomace included moisture content, ash content, fibre content, protein content and fat content, and these were determined using the AOAC standard methods [19]. The total carbohydrate was determined by difference: by subtracting the percentages of protein, fat, fibre, ash and moisture contents from 100. Analyses were carried out in triplicate.

### 2.4. Functional Properties of the Beetroot Pomace–Wheat Composite Flours

The functional properties determined in triplicate on the composite flours included WAC, OAC, SP, solubility, bulk density and dispersibility.

### 2.5. WAC

The WAC of the samples was determined using the procedure by [20]. A 1.0 g of the BPF was preweighed and transferred to a 15-mL dry centrifuge tube (CT). Using a vortex mixer, the flour was mixed with 10 mL of distilled water. The solution was then centrifuged for 15 min at 3500 rpm using a centrifuge (Sorvall RT6000D, DuPont, Connecticut, United Kingdom). After discarding the supernatant, the contents of the tube were reweighed and expressed as grammes of water absorbed per gramme of flour.

 $$\begin{array}{c} \text{WAC }\left(\%\right)=\left(\frac{\left(\text{weight of absorbed water}\right)}{\left(\text{initial weight of composite flour}\right)}\right)\times 100\%. \end{array}$$

### 2.6. OAC

The OAC was calculated using the Bencini method [21]. A 1.0 g of the flour was measured into a 15-mL CT that was preweighed. A 10 mL of sunflower oil was added to the flour, and the mixture was thoroughly mixed using a vortex mixer. The mixture was subsequently made to stand for 30 min. The sample and oil mixture were spun for 20 min at 3000 rpm using a centrifuge (Sorvall RT6000D, DuPont, Connecticut, United Kingdom). The supernatant obtained after the centrifugation process was carefully decanted into a 50-mL measuring cylinder. The volume of the supernatant was subsequently recorded. The CT containing the oil-soaked flour was then weighed. Calculation of the OAC (percentage) was carried out using the formula:

 $$\begin{array}{c} \text{OAC }\left(\%\right)=\frac{\left(\text{weight of CT with absorbed oil by flour}\right)-\left(\text{weight of CT with flour}\right)}{\text{weight of flour sample}}\times 100\%. \end{array}$$

### 2.7. SP and Solubility

The SP and solubility were determined following the method by Oladele and Aina [22]. A 1 g of the BPF was mixed with 10 mL of distilled water in a CT. The mixture was then heated at 80°C for 30 min in a water bath whilst being vigorously shaken. The tube was removed from the bath, wiped dry and centrifuged for 15 min at 2200 rpm after returning to room temperature. Decantation of the clear supernatant into clean, dried preweighed Petri dishes was carefully carried out subsequently. The Petri dishes containing the clear supernatant were heated in the hot air oven at 105°C for 30 min to allow for evaporation. They were subsequently removed, cooled and weighed to determine the mass of the soluble substances in the supernatant. The mass of each flour paste in the CTs determined by difference was also recorded.

$$\begin{array}{cc} \; & \text{Swelling power }\left(\%\right)=\frac{\left(\text{weight of paste}\right)}{\left(\text{weight of sample}\right)}\times 100\%,\\ \text{Solubility }\left(\%\right)=\frac{\left(\text{weight of soluble fraction}\right)}{\left(\text{weight of original sample}\right)}\times 100\%. \end{array}$$

### 2.8. Bulk Density

The Makinde and Ladipo [23] method was followed for the determination of the bulk density of the flour samples. A 10 g of the flour sample was weighed into a 50-mL graduated cylinder. The sample's volume was measured both before and after the cylinder's base was lightly tapped 10 times on a lab bench. A repetition of the procedure was carried out for each sample to ensure uniformity and accuracy. Calculation of the free and tapped bulk densities was subsequently carried out and expressed as grammes per cubic centimetre.

### 2.9. Dispersibility Determination

The dispersibility was calculated using a standard procedure by [24]. About 10 g of the flour sample was transferred into a 100-mL measuring cylinder, and 50 mL of distilled water is then added. The mixture was thoroughly stirred and allowed to sit for 3 h to settle. The volume of settled particles was taken, and %dispersibility was determined as follows:

 $$\begin{array}{c} \text{Dispersibility }\left(\%\right)=\frac{50-\text{volume of settled particle}}{50}\times 100\%. \end{array}$$

### 2.10. Formulation of Rock Buns

The rock buns were formulated from refined wheat flour and in composite preparation with BPF, with the BPF added in increments of 5%. Four distinct types of beetroot rock buns were prepared using wheat flour and BPF in the following ratios: 95:5 for BRB_1_, 90:10 for BRB_2_, 85:15 for BRB_3_ and 80:20 for BRB_4_ with 100% white wheat prepared as the control sample. The list of ingredients used for the rock bun preparation is provided in Table 1.

The other components (composite flours, sugar, baking powder, salt, margarine, milk, eggs and vanilla extract) were combined with baking soda. Ingredients were thoroughly mixed, and the batter was poured into baking pans greased with margarine and baked for 30 min at a temperature of 180°C.

### 2.11. Sensory Evaluation of Rock Buns Formulated From the Wheat and Composite Flours

Ethical approval for sensory evaluation was obtained from the KNUST Committee for Human Research and Publication Ethics (CHRPE/AP/284/24). The liking and sensory acceptability of the beetroot-enriched rock buns were evaluated using consumer sensory testing [25]. A total of 50 untrained panellists (undergraduate students) were recruited from KNUST to evaluate sensory attributes of the rock buns. The attributes evaluated included the acceptance of crumb colour, crust colour, texture, aroma, taste, aftertaste, mouthfeel and overall acceptability and were carried out using a 100 mm unstructured visual analogue scale with anchor points of *extremely like* on the right and *extremely dislike* on the left. The rock buns were presented to the participants in a randomised order on white plates and labelled with three-digit codes. Water was provided to the panellists for palate cleansing before tasting each sample.

### 2.12. Statistical Analyses

All statistical analyses carried out in this study were done using the Statistical Package for Social Sciences (SPSS) software (v24.0). The Shapiro–Wilk test was used to determine the normality of the data. Significant differences in the nutrient composition and functional properties of the composite flours, as well as the sensory characteristics of the beetroot rock buns, were determined using one-way analysis of variance (ANOVA). This was followed by mean comparisons using Tukey's test, where significant differences were established at *p* < 0.05. Principal component analysis (PCA) and agglomerative hierarchical clustering exploiting Ward linkage and Euclidean distances were also used to establish a visual relationship between the flour samples and some of the outcome variables using XLSTAT software [26].

## 3. Result

### 3.1. Proximate Composition of Wheat Flour, Freeze-Dried BPF and Composite Flours

The predominant macronutrient was carbohydrates, comprising 66.8% of wheat flour compared to 57.05% in BPF, and the wheat flour was also significantly higher in moisture and crude fat (Table 2). Conversely, the BPF was higher in ash, crude fibre and protein. Significant differences were observed in the proximate composition of the BPF and the wheat flour. Enrichment of wheat flour with freeze-dried BPF generally resulted in increased moisture content of the composite flours compared to the control wheat flour. Generally, there was an increase in important nutrients including ash, fibre and protein compared to the control following the BPF enrichment. A dose-response increase following the enrichment was observed for the ash and fibre. For the enriched flours, the BRB_4_ recorded the highest ash (2.19%) and fibre (1.79%) content with BRB_1_ recording the least ash (0.85%) and fibre content (1.17%). Carbohydrate content of the composite flours decreased following BPF enrichment compared to the control (Table 2).

### 3.2. Functional Properties of the Wheat and Wheat–Beetroot Composite Flours

Dispersibility of the composite flours decreased with increasing enrichment of the wheat flour with BPF (Table 3). WF had the highest value for dispersibility (47.73%), and BRB_4_ had the lowest dispersibility of 32%. The solubility of the composite flours ranged from 2.87% to 10.39% with WF recording the highest solubility of 10.39% and BRB_4_ recording the lowest value of 2.87%. SP ranged from 500.21% to 540.26% with BRB_3_ having the highest SP (540.26%), whilst BRB_2_ had the lowest (500.21%). The OAC for the flours ranged from 112.37% to 129.93% with BRB_4_ recording the highest value, whilst WF recorded the lowest. WAC ranged from 107.13% for WF to 186.55% for BRB_4_. The loose bulk density values increased with increasing amount of beetroot pomace in the composite flour which ranged from 0.37 g/cm^3^ for WF to 0.43 g/cm^3^ for BRB_4_. For the tapped bulk density, BRB_1_ had the highest value of 0.63 g/cm^3^, whilst BRB_3_ recorded the lowest value of 0.49 g/cm^3^ (Table 3).

**Figure 1:**
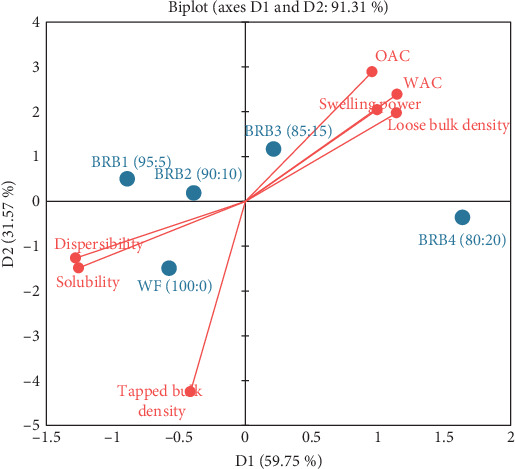
Principal component analysis showing the relationship between the various formulations and their functional properties. WF, 100% wheat flour (control); BRB_1_, 95% wheat flour with 5% beetroot pomace flour; BRB_2_, 90% wheat flour with 10% beetroot pomace flour; BRB_3_, 85% wheat flour with 15% beetroot pomace flour; BRB_4_, 80% wheat flour with 20% beetroot pomace flour.

Samples with high BPF enrichment (BRB_3_ and BRB_4_) were associated with high SP, loose bulk density, WAC and OAC (Figure 1). On the contrary, samples with little to no BPF (WF, BRB_1_ and BRB_2_) reported high dispersibility and solubility.

### 3.3. Sensory Attributes of Rock Buns Formulated From Wheat–Beetroot Pomace Composite Flour

The mean aroma scores of the rock buns ranged from 54.96 to 72.22 with the control sample recording the highest whilst the BRB_3_ recording the lowest (Table 4). In terms of taste, BRB_1_ recorded the highest mean score of 65.16. The mean crumb colour varied from 48.12 to 73.29 with the control having the highest score of 73.29 and the lowest score 48.12 was for BRB_4_. The mean scores for aftertaste ranged from 47.05 to 67.20. The highest mean score for aftertaste was WF, whilst BRB_4_ recorded the lowest. The mean score for texture varied from 54.54 to 66.21 with BRB_1_ recording the highest score and the lowest being BRB_4_. The mean score of mouthfeel ranged from 48.48 to 75.65. The rock bun sample with 5% of beetroot pomace (BRB_1_) had the highest score. The overall acceptability values of the rock bun samples varied from 49.43 to 80.85. The sample with the highest recorded value was WF, whilst the lowest was observed in BRB_4_ (Table 4).

**Figure 2:**
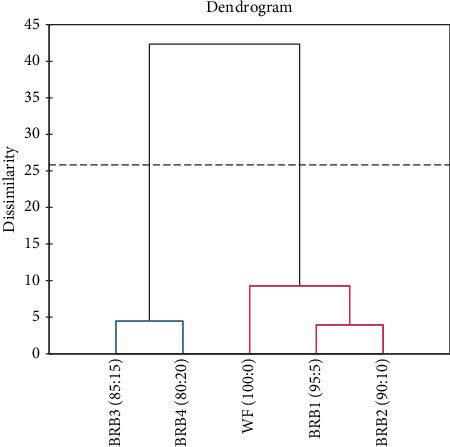
Agglomerative hierarchical clustering for sensory and functional properties. WF, 100% wheat flour (control); BRB_1_, 95% wheat flour with 5% beetroot pomace flour; BRB_2_, 90% wheat flour with 10% beetroot pomace flour; BRB_3_, 85% wheat flour with 15% beetroot pomace flour; BRB_4_, 80% wheat flour with 20% beetroot pomace flour.

The agglomerative hierarchical clustering for sensory and functional properties shows that samples BRB_3_ and BRB_4_ were closely related, whereas BRB_1_ and BRB_2_ were more closely related (Figure 2).

### 3.4. Proximate Composition of the Control Rock Buns and the Most Acceptable Ones

The 5% and 10% freeze-dried BPF-enriched rock buns generally recorded higher nutritional value in the crude fibre compared to the control rock buns (Table 5).

## 4. Discussion

This is the first time freeze-dried beetroot pomace has been used for rock bun enrichment. Enrichment of the wheat-based rock buns with BPF resulted in significant increases in the ash, crude fibre and protein contents. This is consistent with findings reported for plain cakes enriched with oven-dried beetroot pomace (5%, 10%, 15% and 20%) that recorded increased protein (13.01%), crude fibre (55.18%) and ash content (3.57%) compared to the control sample [27]. The utilisation of beetroot powder in cookies has been reported [14]. Similarly, there was an increase in the nutrient profile of the enriched cookies following the BPF enrichment.

Enrichment of the wheat flour with BPF increased its water-holding capacity. Similarly, the incorporation of beetroot into dough increased its water-holding capacity [28]. In that study, the authors reported water-holding capacity of 63.10%, 64.65%, 67.63%, 70.70% and 73.30% for 100% wheat flour and 2%, 5%, 7% and 10% beetroot pomace fibre–wheat flour, respectively, which was similar to this present study. The high WAC could be attributed to the presence of high dietary fibre components with a high number of hydroxyl groups of cellulose within plant tissues which can retain water [29, 30]. The oil holding capacity is a technological characteristic associated with the chemical structure of polysaccharides which is influenced by both their physical and chemical structures such as surface characteristics, thickness, total charge density and the hydrophobic nature of the fibre particles [30, 31]. However, no difference in the oil holding capacity was observed in this study.

Water solubility index predominantly serves as an indicator of molecular component degradation and quantifies the degree of conversion of starch during baking, indicating the amount of soluble polysaccharide released from the starch component following processing [32]. The solubility of the flours ranged from 2.87% to 10.39% with the values decreasing with increasing BPF substitution. The control had the highest solubility of 10.39% which is about five times greater than the lowest solubility observed in BRB_4_. Kumar and Kumar [33] reported a water solubility index of 3.62%, 3.47%, 3.33%, 2.41% and 2.19% for 100% wheat flour and 2.5%, 5%, 7.5% and 10% carrot pomace–wheat composite flour, respectively. Lower solubility values recorded for the composite flours as BPF substitution increased could be attributed to the coagulation of proteins and the amylose-lipid network complex in the flour samples, creating a strong bonding force [34]. These complexes stop amylose granules from leaching, which reduces solubility [34].

The expansion of flours and the porosity of food products can be determined using bulk density. There was an increase in the loose bulk density as the BPF substitution increased in the composite flour. Tap bulk density of the different flours varied from 0.49 to 0.63 g/cm^3^ with the highest tap bulk density recorded for the control and the lowest for the BRB_3_. There was no significant difference between the control and the composite flours for both loose and tap bulk density. Amandikwa et al. [34] reported the tap bulk density of wheat flour to be 0.67% which is similar to the findings in this present study.

In flours, SP impacts on the WAC and thus can be exploited to predict the cooking behaviour of flours and composite flours in food systems [35]. The SP ranged from 500.21% to 540.26%, with BRB_1_ (500.21%) having the lowest, followed by WF (503.9%) and BRB_3_ (540.26%) with the highest SP. However, no significant difference was observed in the SP of the substituted samples in this present study. Dispersibility of the composite flours decreased with increasing beetroot pomace in the flour substitutes. The percentage of dispersibility of the wheat flour and composite flours decreased from 47.73% to 32.00%. BRB_4_ recorded the lowest value of 32.00%, whilst control recorded the highest value of 47.73% for dispersibility. There was no significant difference between the control and BRB_1_. BRB_4_ was significantly different from the control and the other composite flours. Badjona et al. [10] observed an increasing trend of 58%, 59.33%, 60.67%, 61.33% and 65.67% dispersibility for 100% wheat flour, 5% carrot pomace composite flour and 10%, 15% and 20% carrot–pineapple composite flour. This differed from the findings in this present work. The decreasing trend observed for the dispersibility of composite flours could be associated with the reconstruction of bonds formed within the composite flours [17].

The PCA was carried out to provide deeper insight into the relationship between the flour samples and their functional properties. The first and second principal components (D1 and D2) explained 59.75% and 31.57% of the variance, respectively (Figure 1). Moreover, it was found that samples with high composite flour (BRB_3_ and BRB_4_) were associated with high SP, loose bulk density, WAC and OAC. On the contrary, samples with little to no composite flour (WF, BRB_1_ and BRB_2_) reported high dispersibility and solubility (Figure 1). Tapped bulk density appears to play a less important role in explaining differences among the samples. However, these findings are contrary to a work done by [10] on rock buns incorporated with carrot and pineapple pomace. These authors rather reported that samples with high composite flour were associated with high dispersibility, loose density and bulk density, and those with little to no composite flour also reported high OAC, WAC and SP [10]. These contrasting findings could be attributed to several factors such as differences in the components of the composite flours, variability in processing methods and conditions, effects of other additives and interactions with the food matrix.

The sensory results indicate that the overall acceptability of composite samples (BRB_1_ and BRB_2_) was highest among the composite samples and was comparable with the control (WF). The panellists liked the BRB_1_ sample compared to the other formulations (BRB_2_, BRB_3_ and BRB_4_) in terms of aroma, crumb colour, crust colour, texture, taste, aftertaste and mouthfeel. However, the control rock buns were the most accepted by the panellists. Colour is an important factor that has a possible influence on consumers' perception of flavour and acceptability of food products [36]. The colour and appearance of rock buns are a result of the reducing sugars, since these sugars caramelised during baking to give the rock buns their brown colour [37]. The sensory scores for colour decreased with increasing levels of BPF in the composite flours. This was consistent with the research findings by [14] who reported similar observations upon substituting BPF in cookies. The addition of BPF gives the rock buns a brownish-red colour, and this made the rock buns appear darker as the level of freeze-dried BPF increased. The heating of betalain results in the appearance of a light brown colour which causes the rock buns to appear dark [38]. The inner colour of the rock buns was found to be darker compared to the surface colour.

The mean score for taste varied from 54.64 to 65.16. As the amount of beetroot flour in the composite flours increased, the sensory score for taste decreased as a result of the development of a bitter taste caused by the presence of high tannin in the freeze-dried BPF [27].

The mean score of aroma varied from 54.96 to 72.22. The control had the highest value of 72.22 for the aroma, and the values decreased as the freeze-dried BPF proportions increased in the composite flours. Thus, it can be inferred that the incorporation of beetroot flour negatively affected the aroma of rock buns, but BRB_1_ and BRB_2_ were not different from the control.

The mean scores for aftertaste ranged from 47.05 to 66.19. The values decreased as the BPF increased, showing that the incorporation of BPF had a negative influence on aftertaste. The mean score of mouthfeel for rock buns ranged from 48.48 to 75.66. The rock buns with 5% freeze-dried BPF (BRB_1_) had the highest liking for mouthfeel. Similarly, rock buns with 5% freeze-dried BPF recorded the highest value (66.21) for texture. However, a further increase in beetroot flour decreased the liking scores for the texture of the rock buns as evaluated from the sensory evaluation.

In terms of the overall acceptability, the control sample had the highest mean score of 80.85. This was followed by the sample with 5% freeze-dried BPF substitution, with a value of 69.62, and then by the 10% sample. Although the control sample had the highest value, BRB_1_ had the highest value compared to the other rock buns fortified with freeze-dried BPF (10%, 15% and 20%). Therefore, making BRB_1_ the most preferred sample among the samples made with composite flours.

## 5. Conclusion

This study's analysis of the nutritional content of freeze-dried BPF revealed that it had a high concentration of ash and fibre. There was an increase in the fibre, moisture and ash contents of the composite flours as the proportion of freeze-dried BPF increased, whilst fats and carbohydrates decreased. With higher incorporation of freeze-dried BPF, the functional qualities of wheat flour and composite flours—such as their ability to absorb water, oil and their SP—increased, whilst tapped bulk densities, solubility and dispersibility decreased. The composite flour with 20% freeze-dried BPF had significantly (*p* < 0.05) improved nutritional composition as compared to the control sample. The sensory results indicate that the consumer acceptability of the rock buns fortified with 5% and 10% freeze-dried BPF was comparable to the control made solely from wheat flour.

## Figures and Tables

**Table 1:** Ingredients used for rock bun preparation.

**Ingredient**	**Control**	**Wheat flour:beetroot pomace flour**			
	**WF (100:0)**	**BRB**_**1**_ ** (95:5)**	**BRB**_**2**_ ** (90:10)**	**BRB**_**3**_ ** (85:15)**	**BRB**_**4**_ ** (80:20)**
Wheat flour (g)	100	95	90	85	80
Beetroot flour (g)	0	5	10	15	20
Milk (mL)	28	28	28	28	28
Salt (g)	2	2	2	2	2
Margarine (g)	10	10	10	10	10
Baking powder (g)	3	3	3	3	3
Sugar (g)	30	30	30	30	30
Whole egg (g)	40	40	40	40	40
Nutmeg (g)	2	2	2	2	2
Vanilla essence (mL)	1	1	1	1	1

Abbreviations: BRB_1_, 95% wheat flour with 5% beetroot pomace flour; BRB_2_, 90% wheat flour with 10% beetroot flour; BRB_3_, 85% wheat flour with 15% beetroot pomace flour; BRB_4_, 80% wheat flour with 20% beetroot pomace flour; WF, 100% wheat flour (control).

**Table 2:** Proximate composition of the wheat, freeze-dried beetroot pomace and composite flours.

**Parameter (%)**	**WF**	**BPF**	**BRB**_**1**_	**BRB**_**2**_	**BRB**_**3**_	**BRB**_**4**_
Moisture	11.71 ± 0.09^b^	10.54 ± 0.03^a^	12.02 ± 0.08^b^	12.62 ± 0.22^c^	12.89 ± 0.28^b^	12.80 ± 0.14^c^
Ash content	0.63 ± 0.05^a^	9.52 ± 0.09^d^	0.85 ± 0.02^a^	1.36 ± 0.10^b^	1.75 ± 0.33^b^	2.19 ± 0.03^c^
Crude fat	9.50 ± 0.36^d^	4.15 ± 0.29^a^	9.31 ± 0.16^cd^	8.39 ± 0.52^bc^	7.70 ± 0.48^b^	7.40 ± 0.27^b^
Fibre	0.63 ± 0.14^a^	6.65 ± 0.31^c^	1.17 ± 0.18^ab^	1.41 ± 0.39^b^	1.69 ± 0.17^b^	1.79 ± 0.10^b^
Protein	10.73 ± 0.22^a^	12.10 ± 0.20^b^	11.05 ± 0.11^a^	12.04 ± 0.22^b^	11.60 ± 0.22^b^	11.71 ± 0.11^b^
Carbohydrate	66.80 ± 0.42^a^	57.05 ± 0.50^d^	65.60 ± 0.15^b^	64.18 ± 0.74^a^	64.37 ± 0.45^bc^	64.11 ± 0.42^cd^

*Note:* Means with different superscript letters in the same row are significantly different (*p* < 0.05). Values are expressed as mean ± standard deviation of triplicate samples.

Abbreviations: BPF, beetroot pomace flour; BRB_1_, 95% wheat flour with 5% beetroot pomace flour; BRB_2_, 90% wheat flour with 10% beetroot flour; BRB_3_, 85% wheat flour with 15% beetroot pomace flour; BRB_4_, 80% wheat flour with 20% beetroot pomace flour; WF, 100% wheat flour (control).

**Table 3:** Functional properties of wheat and wheat–beetroot composite flours.

**Parameters**	**WF**	**BRB**_**1**_	**BRB**_**2**_	**BRB**_**3**_	**BRB**_**4**_
Dispersibility (%)	47.73 ± 1.42^d^	44.67 ± 2.08^cd^	41.00 ± 1.73^bc^	39.47 ± 2.54^b^	32.00 ± 0.00^a^
Solubility (%)	10.39 ± 2.84^c^	9.17 ± 0.22^bc^	9.72 ± 2.08^c^	4.90 ± 0.69^ab^	2.87 ± 0.25^a^
Swelling power (%)	503.90 ± 67.57^a^	500.21 ± 62.89^a^	507.16 ± 57.83^a^	540.26 ± 94.55^a^	528.90 ± 70.92^a^
Oil absorption capacity (%)	112.37 ± 11.88^a^	123.47 ± 8.41^a^	121.39 ± 14.31^a^	126.22 ± 20.09^a^	129.93 ± 4.86^a^
Water absorption capacity (%)	107.13 ± 12.09^a^	138.48 ± 31.68^ab^	134.09 ± 10.05^ab^	172.09 ± 13.61^bc^	186.55 ± 8.10^c^
Loose bulk density (g/cm^3^)	0.37 ± 0.00^a^	0.38 ± 0.03^a^	0.41 ± 0.01^a^	0.41 ± 0.01^a^	0.43 ± 0.04^a^
Tapped bulk density (g/cm^3^)	0.63 ± 0.06^a^	0.54 ± 0.02^a^	0.54 ± 0.02^a^	0.49 ± 0.10^a^	0.54 ± 0.20^a^

*Note:* Means with different superscript letters in the same row are significantly different (*p* < 0.05). Values are expressed as mean ± standard deviation of triplicate samples.

Abbreviations: BRB_1_, 95% wheat flour with 5% beetroot pomace flour; BRB_2_, 90% wheat flour with 10% beetroot pomace flour; BRB_3_, 85% wheat flour with 15% beetroot pomace flour; BRB_4_, 80% wheat flour with 20% beetroot pomace flour; WF, 100% wheat flour (control).

**Table 4:** Sensory evaluation of wheat and its beetroot pomace flour–enriched rock buns.

**Parameters**	**WF**	**BRB**_**1**_	**BRB**_**2**_	**BRB**_**3**_	**BRB**_**4**_
Aroma	72.22 ± 17.36^b^	69.13 ± 22.96^b^	68.48 ± 21.74^b^	54.96 ± 25.43^a^	63.92 ± 29.87^a^
Taste	62.66 ± 20.92^a^	65.16 ± 22.34^a^	62.73 ± 22.15^a^	54.64 ± 24.30^a^	55.83 ± 95.73^a^
Crumb colour	73.29 ± 16.45^c^	63.77 ± 21.00^bc^	55.20 ± 23.01^ab^	51.53 ± 24.91^a^	48.12 ± 21.56^a^
Crust colour	74.24 ± 18.24^c^	63.56 ± 22.83^bc^	57.26 ± 24.56^ab^	51.36 ± 24.75^ab^	48.65 ± 21.59^a^
Aftertaste	66.19 ± 22.50^c^	67.21 ± 20.03^c^	60.12 ± 27.26^ab^	55.95 ± 25.12^ab^	47.05 ± 24.56^a^
Mouthfeel	61.02 ± 26.20^ab^	75.66 ± 60.25^c^	59.57 ± 22.94^b^	56.48 ± 25.01^a^	48.48 ± 24.20^a^
Texture	65.87 ± 20.93^ab^	66.21 ± 18.73^c^	61.08 ± 22.60^ab^	57.69 ± 21.01^ab^	54.54 ± 21.91^ab^
Overall acceptability	80.85 ± 54.44^c^	69.62 ± 18.94^bc^	64.79 ± 24.68^abc^	55.23 ± 23.96^ab^	49.43 ± 23.41^a^

*Note:* Means with different superscript letters in the same row are significantly different (*p* < 0.05). Values are expressed as mean ± standard deviation of triplicate samples.

Abbreviations: BRB_1_, 95% wheat flour with 5% beetroot pomace flour; BRB_2_, 90% wheat flour with 10% beetroot pomace flour; BRB_3_, 85% wheat flour with 15% beetroot pomace flour; BRB_4_, 80% wheat flour with 20% beetroot pomace flour; WF, 100% wheat flour (control).

**Table 5:** Proximate composition of the control rock buns and the most acceptable ones.

**Parameters (%)**	**WF**	**BRB**_**1**_	**BRB**_**2**_
Ash content	2.82 ± 0.39^a^	2.83 ± 0.62^a^	3.17 ± 0.40^b^
Moisture	22.99 ± 0.52^a^	24.06 ± 0.90^ab^	25.56 ± 0.51^b^
Crude fibre	0.06 ± 0.02^a^	0.57 ± 0.04^b^	1.26 ± 0.21^c^
Crude fat	9.43 ± 0.28^a^	10.34 ± 0.39^a^	10.61 ± 0.72^a^
Protein	13.43 ± 0.03^a^	12.42 ± 0.24^b^	11.28 ± 0.08^c^
Carbohydrate	64.55 ± 0.14^a^	62.04 ± 0.75^b^	59.75 ± 0.59^c^

*Note:* Means with different superscript letters in the same row are significantly different (*p* < 0.05). Values are expressed as mean ± standard deviation of triplicate samples.

Abbreviations: BRB_1_, 95% wheat flour with 5% beetroot pomace flour; BRB_2_, 90% wheat flour with 10% beetroot pomace flour; WF, 100% wheat flour (control).

## Data Availability

The original datasets generated from the study are available from the corresponding author on reasonable request.
